# Is human blood a good surrogate for brain tissue in transcriptional studies?

**DOI:** 10.1186/1471-2164-11-589

**Published:** 2010-10-20

**Authors:** Chaochao Cai, Peter Langfelder, Tova F Fuller, Michael C Oldham, Rui Luo, Leonard H van den Berg, Roel A Ophoff, Steve Horvath

**Affiliations:** 1 Department of Human Genetics, David Geffen School of Medicine, University of California Los Angeles, Los Angeles, CA 90095, USA; 2 Department of Biostatistics, David Geffen School of Medicine, University of California Los Angeles, Los Angeles, CA 90095, USA; 3 Department of Neurology, The Eli and Edythe Broad Center of Regeneration Medicine and Stem Cell Research, University of California San Francisco, San Francisco, CA 94143, USA; 4 Department of Neurology, Rudolf Magnus Institute of Neuroscience, University Medical Center Utrecht, Utrecht 3584 CX, the Netherlands; 5 UCLA Center for Neurobehavioral Genetics, Semel Institute of Neuroscience and Human Behavioral, School of Medicine, University of California Los Angeles, Los Angeles, CA 90095, USA

## Abstract

**Background:**

Since human brain tissue is often unavailable for transcriptional profiling studies, blood expression data is frequently used as a substitute. The underlying hypothesis in such studies is that genes expressed in brain tissue leave a transcriptional footprint in blood. We tested this hypothesis by relating three human brain expression data sets (from cortex, cerebellum and caudate nucleus) to two large human blood expression data sets (comprised of 1463 individuals).

**Results:**

We found mean expression levels were weakly correlated between the brain and blood data (*r *range: [0.24,0.32]). Further, we tested whether co-expression relationships were preserved between the three brain regions and blood. Only a handful of brain co-expression modules showed strong evidence of preservation and these modules could be combined into a single large blood module. We also identified highly connected intramodular "hub" genes inside preserved modules. These preserved intramodular hub genes had the following properties: first, their expression levels tended to be significantly more heritable than those from non-preserved intramodular hub genes (*p *< 10^-90^); second, they had highly significant positive correlations with the following cluster of differentiation genes: *CD58, CD47, CD48, CD53 *and *CD164*; third, a significant number of them were known to be involved in infection mechanisms, post-transcriptional and post-translational modification and other basic processes.

**Conclusions:**

Overall, we find transcriptome organization is poorly preserved between brain and blood. However, the subset of preserved co-expression relationships characterized here may aid future efforts to identify blood biomarkers for neurological and neuropsychiatric diseases when brain tissue samples are unavailable.

## Background

There is no clear consensus regarding the use of blood-based gene expression data for addressing neurological and neuroscientific research questions. On the one hand, gene expression levels in whole blood are only weakly correlated with those in brain tissue [1,2]. On the other hand, blood gene expression profiles have been used to study neuropsychiatric diseases such as bipolar disorder and schizophrenia [3-6], as well as neurological diseases such as Amyotrophic Lateral Sclerosis [7], Huntington's disease [8], Alzheimer's disease [9], and chronic fatigue syndrome [10]. There are at least two major reasons why the relationship between human brain and human blood expression profiles remains poorly understood. The first reason concerns data quality and quantity: it is notoriously difficult to measure human brain tissue expression levels because of potential biases from post-mortem effects and relatively low sample sizes. The second reason is that most previous studies have focused on the preservation of mean expression levels, as opposed to the preservation of co-expression relationships. Given that the human brain transcriptome is organized into biologically meaningful co-expression modules [11-14], it is important to study the preservation of this organization in blood.

Because human brain expression data is derived from post-mortem brain tissue, special attention must be paid to RNA quality, post-mortem interval, and pH. To minimize the influence of these factors, we used highly reproducible and validated brain gene expression data sets from a recent meta-analysis of publicly available brain expression data [12]. Data set 1 (referred to as CTX) consisted of 67 control samples from 67 individuals representing four cortical areas [15-17]. Data set 2 (referred to as CN) consisted of 27 control samples from 27 individuals taken from the head of the caudate nucleus [18]. Data set 3 (referred to as CB) consisted of 24 control samples from 24 individuals taken from cerebellar hemisphere [15].

By applying weighted gene co-expression network analysis (WGCNA) [19-21] to these data sets, Oldham et al. (2008) identified 19 cortex (CTX) modules, 23 caudate nucleus (CN) modules, and 22 cerebellum (CB) modules. These modules were defined as branches of a hierarchical clustering tree and were labeled by different colors. Many modules were highly preserved across the three brain regions, which was why they received the same color label. For example, 45% (*p *= 2.8 × 10^-53^) of genes overlapped between the yellow cortex module (labeled yellow/CTX) and yellow caudate nucleus module (labeled yellow/CN). Similarly, 46% (*p *= 1.1 × 10^-54^) of genes overlapped between the blue/CTX and the blue/CN modules [12]. By considering cell type-specific markers, several brain modules were found to contain genes that are preferentially expressed in oligodendrocytes, astrocytes or neurons [12].

Here we report the results of a comprehensive statistical analysis by cross-referencing the brain expression data with two large blood data sets (comprising a total of 1463 individuals). While previous studies have focused on the preservation of individual gene expression levels across the two tissues, we also investigated the preservation of gene co-expression modules. Since oligodenrocytes, astrocytes, and neurons are not present in blood, we were not surprised that only a handful of human brain modules showed evidence of preservation in human blood. Furthermore, we determined that these preserved modules could be combined into a single large module in blood. We also found that preserved intramodular hub genes tended to have heritable blood expression levels and were highly correlated with a small set of cluster of differentiation (CD) genes.

## Results

### Blood gene expression data

We used whole blood gene expression data from healthy controls in a Dutch data set (n = 405) and published lymphocyte gene expression data (n = 1240), herein referred to as the San Antonio Family Heart Study (SAFHS) data set [22]. The Dutch data set originally consisted of 405 peripheral blood samples from healthy individuals (50.4% men and 49.6% women, mean age 56.4 and range from 19-88). This data set was analyzed with Illumina Human HT-12 microarrays. The SAFHS data set originally consisted of 1240 lymphocytes samples obtained from 1240 individuals (40.8% men and 59.2% women, mean age 39.3 and range from 15-94). This data set was analyzed with Illumina WG-6 microarrays. Using hierarchical clustering with inter-array correlations as a distance measure, we identified potential outlying arrays in an unbiased fashion. Since outlying arrays showed relatively low correlations with the other arrays (across the genes), they were deemed suspicious. To err on the side of caution, we removed these suspicious arrays from the analysis. Potential batch effects (due to different hybridization dates) were also removed using ComBat [23]. These are the same data pre-processing steps that Oldham et al. (2008) used in the brain data analysis. More sample pre-processing information can be found in Additional file 1.

After these pre-processing steps, 380 samples remained in the Dutch data set and 1084 samples remained in the SAFHS data set. Multiple probes corresponding to one gene (symbol) were combined into one measurement. Next, we merged the Affymetrix (brain) data with the Illumina (blood) data by gene symbol, which resulted in 8799 genes in each data set.

### Preservation of mean expression levels and connectivity

We first studied the preservation of mean gene expression levels of the 8799 genes between brain and blood. The pairwise scatterplots in Additional file 2 related mean expression values in the three brain regions to mean expression values in the two blood data sets. We found significant but weak correlations (*r *range: [0.24,0.32]) between mean expression in brain and mean expression in blood.

Next we investigated the extent to which co-expression patterns were preserved between brain and blood. For each gene, the network connectivity (also known as degree) is defined as the sum of its connection strengths with all other genes in the network. Thus, connectivity measures how correlated a gene is with all other genes (see Methods). Genes with high connectivity are informally referred to as "hub" genes. Overall, we found that gene connectivity was even less preserved (*r *range: [0.021,0.11], Additional file 3) in blood than mean expression levels. These results show that global co-expression relationships are poorly preserved between brain and blood. However, Additional file 3 also shows some genes with high connectivity in both data sets. These genes may be part of sets of genes (co-expression modules) that are preserved between the two tissues. A more focused analysis that considered individual modules did reveal some evidence of preservation between the two tissues, as described below.

### Preservation of brain co-expression modules in blood

Oldham et al. (2008) applied rigorous gene filters to the brain data set to ensure that transcripts were present and had high connectivity in the brain data (see the Supplemental Information of Oldham et al. 2008). These filters reduced the number of probe sets in each network to 5549 (CTX), 4050(CN), and 4029 (CB). After combining probes into single measures for each gene symbol and merging the data with the blood data sets, the CTX network contained 2640 genes, CN network contained 2063 genes and the CB network contained 2001 genes.

To determine whether a module found in a reference data set (e.g. human cortex) can also be found in a test data set (e.g. the Dutch blood data set), we used a powerful module preservation statistic approach implemented in the R software function modulePreservation [20] (described in Methods). This permutation test procedure evaluates whether module genes show significant evidence of network connectivity preservation in the test data. This module preservation test results in a statistic (referred to as preservation Z statistic or Zsummary statistic) for each module. The higher the preservation Z statistic is for a given brain module, the stronger the evidence that the brain module is preserved in a given blood data set. Under the null hypothesis of no module preservation, the preservation Z statistic follows an approximately standard normal distribution. Comprehensive simulation studies led to the following thresholds: a module shows no evidence of preservation if its Z statistic is smaller than 2; a Z statistic larger than 5 (or 10) indicates moderate (strong) module preservation.

**Figure 1:**
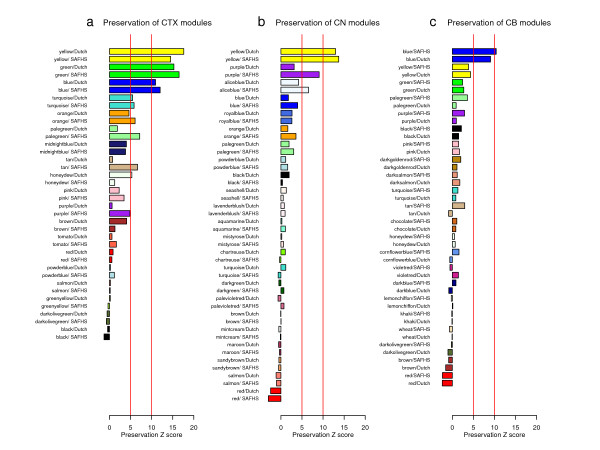
**Studying the brain module preservation in human blood**. The row bars correspond to brain co-expression modules found by Oldham et al. (2008). Modules are labeled by a color. For each module color, there are two horizontal bars which correspond to the module preservation Z statistics in the Dutch blood data and the SAFHS blood data, respectively. The two red vertical lines correspond to thresholds of moderate preservation (5) and strong preservation (10). Panel (a) shows that only three (yellow, green, and blue) out of 19 cortex modules showed strong preservation in both blood data sets. Panel (*b*) shows that only one (yellow) out of 23 caudate nucleus modules was strongly preserved in both blood data sets. Panel (c) shows that only one (blue) out of 22 cerebellum modules was strongly preserved in both blood data sets. In summary, only five modules from Oldham et al. (2008) show strong evidence of preservation in human blood.

We started out by evaluating the preservation of CTX modules in the Dutch and SAFHS blood data sets. The horizontal barplots in Figure 1a show that the preservation Z statistics of the blue, yellow, and green CTX modules were above the threshold of 10 in both blood data sets, i.e. these modules showed strong evidence of preservation. Similarly, Figure 1b presents the module preservation results for the CN modules identified by Oldham et al. (2008). Only the yellow CN module was strongly preserved in both blood data sets. Figure 1c shows that only the blue CB module was strongly preserved in both blood data sets. In total, we find that five brain modules were strongly preserved in human blood. More details and numeric values are presented in Additional file 4.

The preserved modules tended to be relatively large: Out of 2640 CTX network genes (from merging the CTX data with the blood data), 690 were part of the blue module, 421 were part of the green module and 658 were part of the yellow module. The preserved (yellow) CN module contained 254 genes out of 2063 CN network genes. The preserved CB (blue) module contained 819 out of 2001 CB network genes. Thus, 67% of genes in the cortex network, 12% of genes in the caudate nucleus network, and 41% of genes in the cerebellum network were part of a preserved module.

One can also visualize the evidence of module preservation by clustering the genes in the blood data sets. Since the brain modules were defined as branches of a hierarchical clustering tree (dendrogram), we used the identical approach to define modules in the blood gene expression data. Additional file 5 shows dendrograms of the blood gene expression data. As described in the Methods section, blood modules were defined as branches of the dendrogram [20,21]. The first color-band underneath each dendrogram encodes blood module colors. The remaining color bands indicate module membership in brain modules. Visual inspection of these dendrograms revealed that genes from the preserved modules (based on the permutation test) tended to cluster together in the blood data. The fact that some colors were not contiguous shows that the preservation is not perfect. Below, we define measures of module membership to identify the subsets of genes inside each of the five preserved modules that showed the strongest evidence of preservation.

### Relationships among preserved modules in blood

While the brain modules were clearly distinct in the brain data sets, their preserved counterparts no longer appeared distinct in the blood data sets. To explore the relationships among preserved modules in blood, we summarized module expression profiles by forming the first principal component, which is referred to as the module eigengene (ME) [21,24,25]. For example, ME(blue/CTX) denotes the module eigengene of the blue cortex module. The ME can be considered a weighted average of the gene expression profiles in a module. If the ME of one module is highly correlated with that of another module in the blood data, then the genes inside the two modules have similar blood expression patterns, i.e. the two modules cannot be distinguished.

**Figure 2:**
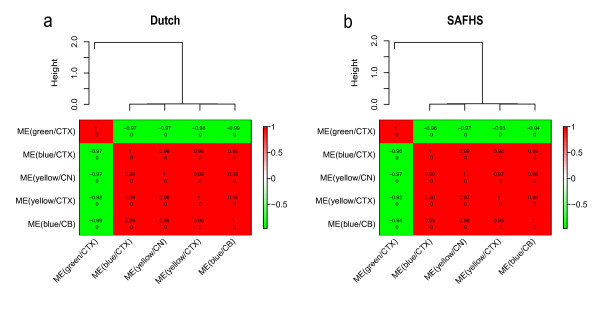
**Relationships between the five preserved modules in the two blood data sets**. The expression profiles of each preserved module were summarized by the respective module eigengene (defined as the first principal component). The correlations between the module eigengenes can be used to measure relationships between the modules (Langfelder and Horvath 2007). The hierarchical cluster tree shows the correlation relationships between the module eigengenes in the Dutch blood data (a) and the SAFHS blood data (b). The tables show the pairwise correlation coefficients (upper number) between the eigengenes and the correlation test p-values (lower number). The colors of the table entries color code the values of the correlations (green and red correspond to negative and positive correlations). Note that the four modules ME(blue/CTX), ME(yellow/CN), ME(yellow/CTX), and ME(blue/CB) were highly positively correlated with each other but negatively correlated with ME(green/CTX).

For the Dutch data set and the SAFHS data set, Figure 2 shows that ME(blue/CTX), ME(blue/CB), ME(yellow/CTX), and ME(yellow/CN) had highly significant positive correlations (*r *> = 0.95, *p *< = 10^-40^) with each other, but highly significant negative correlations (*r *< = -0.95, *p *< = 10^-40^) with ME(green/CTX). This result indicates that the five preserved brain modules can hardly be distinguished in an unsigned gene co-expression network in blood, as they coalesce into one large preserved module. It is natural to ask whether these five modules were distinct in the original brain data sets. Additional file 6 shows that the three preserved CTX modules (blue/CTX, green/CTX, and yellow/CTX) were only moderately correlated in the CTX data: the correlation between ME(blue/CTX) and ME(yellow/CTX) was 0.52; the correlation between ME(blue/CTX) and ME(green/CTX) was -0.09; the correlation between ME(yellow/CTX) and ME(green/CTX) was -0.69. The brain data did not allow us to correlate MEs of different brain regions, since the data consisted of samples from different individuals.

### Preservation of module membership between brain and blood

We defined a measure of module membership (MM) by correlating the ME with each gene expression profile [26]. For example, MMblue_i _= cor(x_i_, MEblue) measures how correlated the expression profile of the i-th gene is with the blue ME. If MMblue_i _is close to 0, the i-th gene is not part of the blue module. But if MMblue_i _is close to 1 (or -1), it is highly positively (or negatively) correlated with the blue module genes. The module membership measure is highly related to intramodular connectivity [26]; thus, a gene with high absolute value MMblue_i _turns out to be a highly connected hub gene inside the blue module.

**Figure 3:**
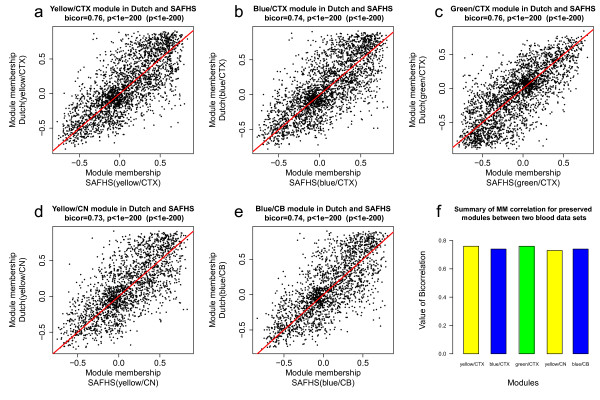
**Module membership measure of preserved modules is highly reproducible between the two blood data sets**. For each of the five preserved brain modules, the scatterplots show that module membership measure was reproducible between the SAFHS blood data (x-axis) and the Dutch blood data (y-axis). Each dot corresponds to a gene. The red diagonal line corresponds to y = x. Results are shown for the following preserved modules: (a) yellow/CTX, (b) blue/CTX, (c) green/CTX, (d) yellow/CN module, and (e) blue/CB. We report both uncorrected correlation test p-values and Bonferroni corrected p-value (inside of brackets). The extremely significant correlation test p-values reflect the large sample size (number of gene). It may be more meaningful to focus on the correlation coefficients. Overall, we find that the module membership measures are highly reproducible.

For each of the five preserved modules, we defined module membership measures in the respective brain data set and the two blood data sets (Additional files 7, 8 and 9). Figure 3 shows that MM measures were highly correlated between the two blood data sets, indicating that the MM measure can be robustly defined in blood.

The extremely significant correlation test p-values in the scatterplots reflect the large sample size, i.e. numbers of genes. It may be more meaningful to consider the correlation coefficient value, e.g. a correlation value of 0.76 indicates a strong (but not perfect) linear relationship. We combined the MM measures for the Dutch and SAFHS data to arrive at a summary measure for human blood, which was referred to as "Blood MM measure". Additional file 10 reports the correlations between the summary blood MM measure and the two individual blood MM measures.

**Figure 4:**
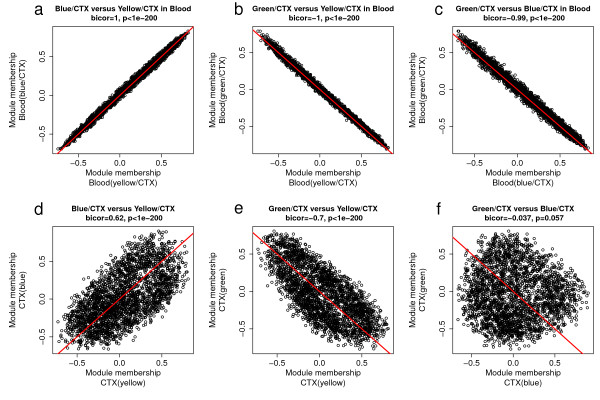
**Relationships between the module membership measures of the three preserved cortex modules**. Relationships between the module membership of blue/CTX, yellow/CTX and green/CTX modules in blood (a-c) and in the original cortex brain samples (d-f). Panel (a) shows that the correlation between MMyellow and MMblue equaled 1 in the blood data, which reflects the fact that these modules were indistinguishable in blood. In contrast, panel (d) shows that correlation between MMyellow and MMblue equaled 0.62 in cortex. Figure (b-c) shows that MMgreen had a correlation close to -1 with MMyellow (b) and MMblue (c) in blood. We report both uncorrected correlation test p-values and Bonferroni corrected p-value (inside of brackets). Given the very high correlations between MMgreen, MMyellow, and MMblue in blood, we combined the three measures in an overall module membership measure referred to as MM.combined.Blood. Analogously, we combined the three MM measures for the cortex network (referred to as MM.combined.CTX).

Figure 4 shows that MM values for the three preserved CTX modules (yellow/CTX, green/CTX, and blue/CTX) were highly correlated in the blood data sets, which reflects what we already know from our eigengene-based analysis (Figure 2): these modules are indistinguishable in blood. Specifically, MM of yellow/CTX was positively correlated with MM of blue/CTX (|*r*| = 1, p < 10^-200^, Figure 4a), while MM of green/CTX was negatively correlated with MM of both yellow/CTX and blue/CTX (Figure 4b-c). Given the exceptionally high correlations between the individual MM measures, it made sense to combine them by forming a weighted average, which flipped the sign of the negatively related green CTX module. We refer to the weighted average MM (across the modules) as the "combined MM measure". Additional file 11 shows that the combined MM value was highly correlated with the original MM value from the three modules.

**Figure 5:**
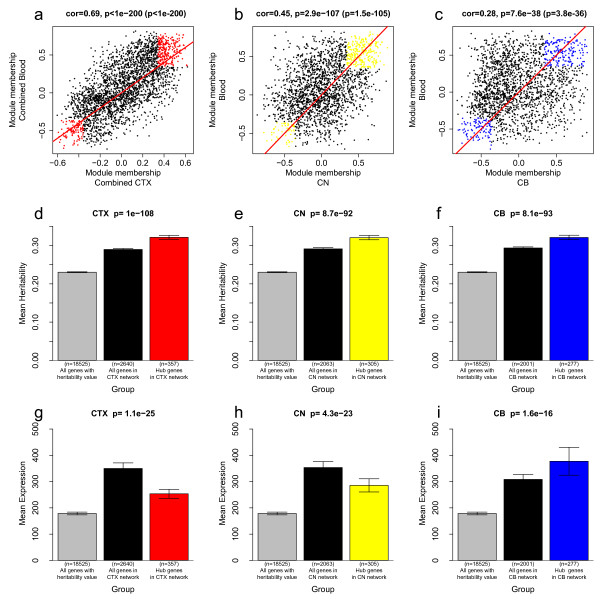
**Definition and characterization of preserved intramodular hub genes**. The scatterplots show how the combined measure of module membership in blood MM.combined.Blood (or MM.Blood, y-axis) related to the analogous measure in cortex (a), caudate nucleus (b), and cerebellum (c). Preserved intramodular hub genes were defined as those genes whose combined MM measure in blood and brain tissue is larger than +0.35 (or smaller than -0.35), since these genes showed highly significant evidence of being part of the preserved modules in both tissues. For the CTX, CN, and CB networks, the roughly 300 preserved intramodular hub genes are colored in red (a), yellow (b), and blue (c). We report both uncorrected correlation test p-values and Bonferroni corrected p-value (inside of brackets). Barplots (d-f) show the mean heritability of the blood expression profiles (y-axis) across preserved intramodular hub genes (blue bars), across genes in the CTX (d), CN (e), and CB (f) networks (black bars), and across all genes on the blood array (grey bar). Note that the preserved intramodular hub genes have significantly higher mean heritability than non-preserved intramodular hub genes. Barplots (g-i) show the mean blood expression values (y-axis) across the same groups of genes. Note that the preserved intramodular hub genes and the brain network genes have significantly higher mean expression values in blood than all genes on the array (grey bar). However, preserved intramodular hub genes do not have higher mean expression levels than the (roughly 2500) genes that form the brain network (black bars).

Although the three modules were distinct in the cortex data, their MM measures also showed high correlations in cortex (Figure 4d-f), which allowed us to define a combined MM measure for the CTX data. The combined cortex MM measure was significantly correlated (*r *= 0.69, *p *< 10^-200^, Figure 5a) with the combined blood MM measure. At the same time, the CN MM measure and the CB MM measure also showed significant correlations with the blood MM measure (*r *= 0.45, *p *< 2.9 × 10^-107^, Figure 5b; *r *= 0.28, *p *< 7.6 × 10^-38^, Figure 5c). These results support the original finding that the five co-expression modules (blue/CTX, green/CTX, yellow/CTX, yellow/CN and blue/CB) exhibit significant preservation in blood.

### Definition of preserved intramodular hub genes

We refer to genes with high module membership in a preserved module as a preserved intramodular hub gene. Preserved hub genes show highly significant evidence of being centrally located inside a preserved module. Specifically, we defined preserved CTX module hub genes as having consistently high positive or negative combined MM in both cortex and blood. Toward this end, we thresholded the combined MM measures in both blood and cortex at a value of +0.35 and -0.35 (corresponding to a correlation test *p*-value < 5 × 10^-13 ^in blood). These thresholds resulted in 357 preserved CTX hub genes, which are colored in red in Figure 5a. For the preserved yellow CN module and preserved blue CB module, we found 305 preserved CN hub genes (colored yellow in Figure 5b) and 277 preserved CB hub genes (colored blue in Figure 5c) using the same threshold.

In summary, only 357 genes (13.5%) from the CTX network, 305 genes (14.8%) from the CN network and 277 genes (13.8%) from the CB network are preserved intramodular hub genes. These preserved intramodular hub genes exhibited the following overlap: the sets of preserved CTX (357) genes and preserved CN (305) genes shared 123 genes (Fisher's exact *p*-value < 2.2 × 10^-16^). The sets of preserved CTX (357) genes and preserved CB (277) genes shared 109 genes (Fisher's exact *p*-value < 2.2 × 10^-16^). The sets of preserved CN (305) genes and preserved CB (277) genes shared 64 genes (Fisher's exact *p*-value < 1.8 × 10^-15^). All three sets of preserved intramodular hub genes shared 36 genes. The names of these preserved hub genes and their MM values can be found in Additional file 12. The biological role of the 36 genes is discussed below.

**Figure 6:**
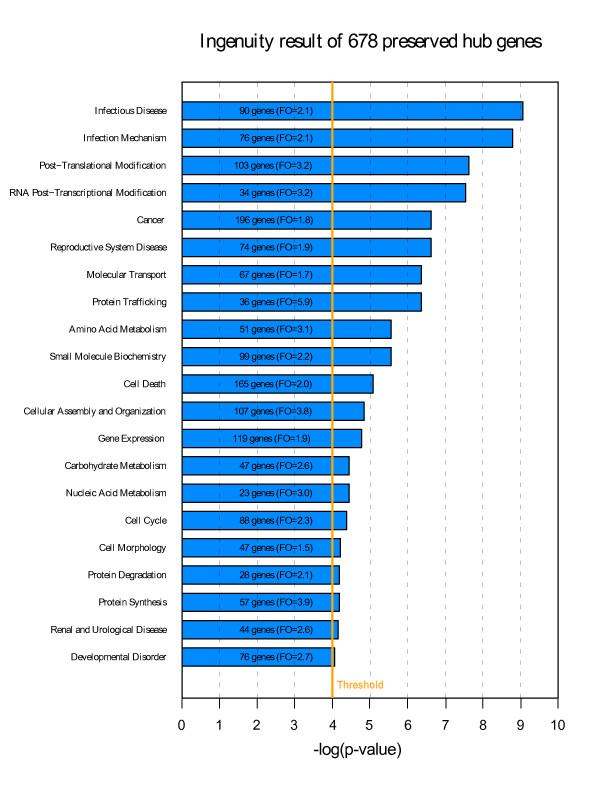
**Ingenuity analysis result for 678 preserved hub genes**. Functional enrichment analysis of the preserved intramodular hub genes found in CTX, CN or CB data (selected in Figure 5) (678 genes). Only gene categories with significant enrichment p-values are presented. FO (inside brackets) denotes fold overrepresentation (defined as observed counts divided by expected counts under the null hypothesis). To calculate the Fisher's exact p-values and FOs, we use the Ingenuity default background (here all human genes).

The union of the three sets of preserved intramodular hub genes contains 678 genes. A functional enrichment analysis of the 678 genes reveals that some of these preserved hub genes play a role in infectious disease and infection mechanism (*p *= 8.6 × 10^-10^), post-translational modification (*p *= 2.4 × 10^-8^), and RNA post-transcriptional modification (*p *= 2.9 × 10^-8^) (Figure 6). A more detailed functional enrichment analysis for each set of preserved CTX, CN, and CB module genes can be found in Additional file 13.

### Preserved intramodular hub genes have more heritable expression levels

In the original publication of the SAFHS data, the authors calculated the heritability of each gene expression level and created a heritability table [22]. The gene expression heritability measures the proportion of expression trait variance attributable to genetic variance. These data allowed us to test whether preserved intramodular hub genes have more highly heritable expression levels than non-preserved intramodular hub genes. The red, yellow and blue bars in Figure 5d-f show the mean heritability (y-axis) for the preserved CTX, CN and CB hub genes, respectively. To facilitate a comparison, we also report the mean heritability for all genes in heritability table (from Goring et al. 2008, grey bars) and for all genes in the merged blood and brain data set (black bars).

Figure 5d shows that that the preserved CTX hub genes (n = 357, red bar) have a significantly (analysis of variance test *p *= 10^-108^) higher mean heritability (32%) than all genes in heritability table (n = 18525, mean heritability: 23%) and all genes in the CTX network (n = 2640, mean heritability: 29%). Analogous results were observed for the CN data set (*p *= 8.7 × 10^-92^, Figure 5e) and the CB data set (*p *= 8.1 × 10^-93^, Figure 5f). These differences in heritability did not reflect differences in mean expression levels, as can be seen from Figure 5g-i, which report mean blood expression values (y-axis) across the different groups of genes. While preserved intramodular hub genes and brain network genes had significantly higher mean expression values than all genes in the heritability table (grey bar), preserved intramodular hub genes did not have higher mean expression levels than brain network genes (black bars).

### Relationships between preserved modules and cluster of differentiation genes

We also investigated the relationships between the preserved modules and a special class of cell surface markers: cluster of differentiation (CD) genes, which are routinely used to characterize blood cell types. If a module is enriched with cell type-specific genes, then its module eigengene should have a strong correlation with the expression values of CD genes that are specific to that cell type. A high positive correlation would therefore suggest that a particular cell type might be related to the module. We found that the MEs of the five preserved modules had highly significant (*p *< 10^-40^) positive correlations with the following CD genes: *CD58, CD47, CD48, CD53 *and *CD164*. Statistical details for the individual modules are presented in Additional file 14.

In the following, we briefly describe what is known about the products of these CD genes while Additional file 15 presents more detailed gene information (adapted from http://www.genecards.org and http://pathologyoutlines.com).

*CD58 *(present on Antigen Presenting Cells) is known to be a ligand of the T lymphocyte CD2 protein, and functions in adhesion and activation of T lymphocytes.

*CD47 *(present on leukocyte, neuroblast, glial cell and other cells) is a membrane protein, which is involved in the increase in intracellular calcium concentration that occurs upon cell adhesion to extracellular matrix.

*CD48 *(present on lymphocyte and other cells) is an activation-associated cell surface glycoprotein, and involved in facilitating interaction between activated lymphocytes.

*CD53 *(present on leukocyte, glial cell and other cells) is cell surface glycoprotein and involved in the regulation of cell development, activation, growth and motility.

*CD164 *(present on leukocyte, glial cell and other cells) is a type I integral transmembrane sialomucin that functions as an adhesion receptor. It is involved in hematopoiesis, migration of umbilical cord blood, prostate cancer metastasis, infiltration of bone marrow, myogenesis and myoblast migration.

### Module preservation between different brain regions

As mentioned in the introduction, many brain modules were found to be highly preserved across the three brain regions, which is why they received the same color label. Here we use a more powerful approach for measuring module preservation (based on the modulePreservation R function) than the one used in the original analysis by Oldham et al. Therefore, we use the modulePreservation function to re-analyze brain module preservation across brain regions. For example, we evaluate which CTX brain modules are preserved in the CN and CB data. Detailed results of this analysis can be found in Additional file 16. For CTX brain modules, we find that 11 out of 19 CTX module show at least moderate evidence of preservation (Preservation Z statistic > 5) in both CN and CB data. For CN brain modules, we find that 12 out of 23 CN modules also show at least moderate evidence of preservation (Preservation Z statistic > 5) in both CTX and CB data. For CB brain modules, we find that 12 out of 22 CB modules show at least moderate evidence of preservation (Preservation Z statistic > 5) in both CTX and CN data. In summary, 55% modules showed preservation cross the different brain regions. These results are congruent with those presented in the original analysis by Oldham et al. It is particularly interesting to study which of our 5 preserved blood/brain modules are preserved in other brain regions.

For the 3 preserved CTX/blood modules (blue, green and yellow), we find that all 3 of them showed very high evidence of preservation in both CN (Preservation Z statistic > = 16.6) and CB data (Preservation Z statistic > = 8.7).

For the preserved (yellow) CN/blood module, we find very high evidence of preservation in CTX data (Preservation Z statistic = 19.1), but only moderate/weak evidence preservation in CB data (Preservation Z statistic = 3.8).

For the preserved (blue) CB/blood module, we find very high evidence of preservation in both CN (Preservation Z statistic = 20.8) and CB data (Preservation Z statistic > 16.0). Further, details can be found in Additional File 16.

Overall, we find strong evidence that the preserved brain/blood modules are also preserved in multiple brain regions.

## Discussion

Few studies are able to access human neural tissue for studying diseases [27]. Given the difficulty of procuring human brain tissue versus the relative ease of measuring blood expression levels, a question of great practical importance is to determine to what extent blood is a reasonable surrogate for brain in gene expression studies. Here we relate highly reproducible brain expression data from a recent meta-analysis of human brain data sets to two large blood data sets. Overall, we find that mean expression levels are weakly preserved between three brain regions and blood (*r *range [0.24,0.32]). Since gene expression profiles in human brain regions are organized into highly reproducible co-expression modules [12], it is important to determine which of these modules show evidence of preservation in blood. Only three out of 19 cortex modules, one out of 23 caudate nucleus modules and one out of 22 cerebellum modules show strong evidence of preservation. In blood, these five modules exhibit very similar expression patterns as can be seen from the very high absolute correlations (|*r*| > 0.96) between their respective eigengenes (Figure 2).

Although few modules were preserved, they tended to be relatively large. 67% of genes in the cortex network were part of one of the three preserved modules; 41% of genes in the cerebellum network and 12% of the caudate nucleus network genes were part of their respective preserved modules. Intramodular hub genes inside preserved modules are centrally located in both modules. The number of intramodular hubs depends on the threshold used for the module membership measures in brain and blood. 13.5% (357) of genes in the cortex network, 14.8% (305) of genes in the caudate nucleus network, and 13.8% (277) of genes in the cerebellum network were defined as preserved intramodular hub genes. Using our posted data and R software code, the reader can change the thresholds used for defining these hub genes. Our biological characterization of preserved intramodular hub genes is highly robust with respect to the chosen threshold values.

In mice, mean expression levels of heritable genes have been found to be highly correlated between mouse hippocampus and spleen [28]. We do not find that heritable genes exhibit highly correlated mean expression levels between brain and blood (Additional file 17). However, we find that the preserved intramodular hub genes tend to be more heritable (Figure 5).

The preserved CTX blue, green, and yellow modules were found to be enriched with neuronal markers, glutamatergic synapse genes, and metabolism-related genes, respectively. The preserved CN yellow module was also found to be enriched with metabolism-related genes, while the preserved CB blue module was enriched with neuronal markers and genes encoding synaptic proteins [12]. In blood, studying the enrichment with regard to brain cell type markers is not meaningful. However, one can classify blood cell types using human clusters of differentiation (CD) genes. Interestingly, the following CD molecules consistently have significant positive correlation with genes inside the preserved modules: *CD58*, *CD47*, *CD48*, *CD53 *and *CD164*.

A functional enrichment analysis of brain module preservation reveals basic functional pathways preserved between the two tissues. Figure 6 shows that these preserved intramodular hub genes are significantly enriched for genes that play a role in infectious disease and infection mechanism, post-translational modification and RNA post-transcriptional modification. Other categories include Cell Death, Energy Production, Nucleic Acid Metabolism, Molecular Transport and Protein Trafficking (Figure 6). The 36 intramodular hub genes that were preserved in all three sets exhibit several common functional themes. First, nearly 20% of these genes, including *ASF1A*, *ATF2*, *DR1*, *HCFC1R1*, *HMGN4*, *MBD3*, and *RAD21*, are known to play roles in modifying chromatin structure. Some of these modifications have been shown to induce transcription (e.g. *ATF2*, *DR1*, *HMGN4*), while others produce repressive effects (e.g. *MBD3*). A number of other genes in the group of 36 encode signalling proteins that are thought to play roles in a wide variety of cellular processes, including *ARPP-19*, *CSNK1G3*, *MAP4K5*, *PPP1CB*, and *YWHAQ*. A third category of genes relates to protein trafficking and includes *RAB1A*, *SNX2*, *SNX3*, while a fourth category consists of genes involved in mitochondrial function, including *DLAT*, *SUCLA2*, and *YME1L1*. Some of the proteins encoded by these 36 genes may physically interact, such as ATP6AP2, which associates with the transmembrane sector of vacuolar ATPases (proton pumps), and ATP6V1C1, which is a subunit of the vacuolar ATPase protein complex. Intriguingly, for a number of other genes in this group, biological functions remained to be elucidated (e.g. *FAM3C*, *FLJ20254*, *LANCL1*, *PRNP*, *RABGGTB*, and *WRB*). We note that many of these 36 preserved intramodular hub genes are expressed ubiquitously. Therefore, it is possible, perhaps even probable, that these genes are also co-expressed in other tissue types beyond brain and blood. Their co-expression may therefore help serve to maintain differentiated cells in a particular state (e.g. chromatin modifying genes) in response to a particular environment (e.g. signalling genes), as well as enable other shared, basic cellular processes (e.g. protein trafficking, energy metabolism).

Our study has several strengths including the use of multiple large data sets, carefully validated brain co-expression modules from Oldham et al 2008, and a powerful statistical approach for evaluating module preservation.

But our study also has several limitations including the following. First, the brain expression data were measured using the Affymetrix platform, while the blood expression data were measured using the Illumina platform. Since platform differences bias our results towards the null hypothesis of no preservation, we can be confident about preservation, but less confident about lack of preservation. The weak correlations between mean expression profiles may reflect platform differences. A second limitation is that we studied the preservation of brain modules in blood (and not vice versa). Our goal was to determine the preservation of robustly defined and well annotated brain modules. Defining blood modules and studying their preservation in brain tissue is beyond the scope of this article. A third limitation is the relatively small set of genes considered for the co-expression module preservation study. Oldham et al. had applied stringent filtering criteria to construct the brain network, which greatly reduced the number of probes considered in that study. After combining probes by gene symbol and merging the brain and blood data, the co-expression module preservation study focused on 2604 CTX, 2001 CB, and 2063 CN network genes. We focused on this relatively small set of genes since their connectivity pattern in brain was found to be highly reproducible across array platforms and independent data sets (Oldham et al 2008). But we should point out that our study of mean expression preservation involved 8799 genes. A fourth limitation is that we only use correlation network methodology. Many alternative co-expression network methods have been proposed in the literature [27,29,30]. We focus on WGCNA since i) this method was used in Oldham et al (2008), ii) it is highly robust [19,21], and iii) it affords a geometric interpretation of network concepts [26,31]. An exploration of alternative procedures is beyond our scope but we encourage the reader to apply their method to our posted data.

## Conclusions

In summary, we find that transcriptome organization is poorly preserved between brain and blood and only a handful of large brain co-expression modules that exhibit strong overall evidence of preservation in blood. However, these modules are not preserved whole cloth. Instead, only certain aspects of these modules (i.e. subsets of genes appear to be involved in basic cellular processes, such as metabolism) exhibit strong preservation of gene co-expression relationships. The subset of preserved co-expression relationships characterized here may aid future efforts to identify blood biomarkers for neurological and neuropsychiatric diseases when brain tissue samples are unavailable.

## Methods

### Weighted gene co-expression network analysis and preservation visualization

The statistical analysis software (WGCNA R package) and R tutorials for constructing a weighted gene co-expression network can be found in [20]. The WGCNA package first calculates all pairwise Pearson correlations coefficients across all samples. In a weighted network, the resulting Pearson correlation matrix is transformed into a adjacency matrix (a_ij _= |cor(x_i_, x_j_)|^*β*^), which represent the pairwise connection strengths. Here we use an unsigned network which ignores the sign of the corrlation relationship since this approach was used in the orignal brain data analysis [12]. However, we mention that one can also construct signed weighted networks that keep track of the sign of the correlation [32]. The power *β *facilitates a soft-thresholding approach that preserves the continuous nature of the co-expression relationships [19-21]. As a power we chose the default value of 6. An advantage of weighted networks is that they are highly robust with regard to the choice of the soft threshold parameter value. As a network dissimilarity measure we used 1 - the topological overlap measure as input for average linkage hierarchical clustering. The topological overlap measure is a highly robust measure of interconnectedness [33,34]. We used the dynamic branch cutting method to define modules as branches of the hierarchical clustering tree [35]. Unassigned background genes, outside of each of the modules, were denoted with the color grey.

### Connectivity and module membership measures

Whole network connectivity for a certain gene is defined as the sum of its connection strengths with all other genes in the network. Mathematically, it can be calculated easily as the sum of a given column in the adjacency matrix. Intramodular connectivity is defined as the sum of the connection strengths between a particular gene and all other genes in the same module. Module membership (MM), or eigengene-based connectivity (kME), is another measure of connectivity. It is defined as MM^q^_i _= cor(x_i_, ME^q^), where x_i _is the expression profile of i-th gene and ME^q ^is the eigengene of q-th module. The larger the absolute values of MM, the greater the similarity between the i-th gene and the q-th ME. If the absolute value of MM is 0, it means that this gene is not part of the module. Although the MM measure is highly correlated with intramodular connectivity [26], the MM measure is preferred since it can be easily extended to genes outside the original module, and the statistical significance (*p*-value) of MM can be calculated for every gene with respect to every module.

### Correlation tests

To measure the relationship between brain tissue connectivity and blood tissue connectivity (and for relating mean expressions), we used a robust estimator of the correlation (the biweight midcorrelation implemented in the WGCNA R package) to protect against outliers. Simulation studies show that the biweight midcorrelation is more robust than the Pearson correlation but often more powerful than the Spearman correlation.

### Correcting p-values for multiple comparison tests

To protect against false positives due to multiple testing, we also report Bonferroni corrected p-values. The Bonferroni correction method is the most conservative approach for correcting for multiple comparisons. The corrected p-value is defined by the product of the uncorrected p-value times the number of tests. Since we carried out 50 correlation tests in this article, a Bonferroni corrected p-value is defined by multiplying the uncorrected p-values by 50.

### Module Preservation analysis

Our module preservation analysis is based on the modulePreservation R function implemented in the WGCNA R package. The modulePreservation function implements several powerful network based statistics for evaluating module preservation. These statistics are summarized into the composite preservation called Zsummary. For each module in the reference data (e.g. brain data) one observed a value Zsummary in the test data (e.g. a blood data set). An advantage of the preservation Z statistic is that it makes few assumptions regarding module definition and module properties. Traditional cross-tabulation based statistics are inferior for the purposes of our study. While cross-tabulation approaches are intuitive, they have several disadvantages. To begin with, they are only applicable if the module assignment in the test data results from applying a module detection procedure to the test data. Even when modules are defined using a module detection procedure, cross-tabulation based approaches face potential pitfalls. A module found in the reference data set will be deemed non-reproducible in the test data set if no matching module can be identified by the module detection approach in the test data set. Such non-preservation may be called the weak non-preservation: "the module cannot be found using the current parameter settings of the module detection procedure''. On the other hand, here we are interested in establishing strong non-preservation: "the module cannot be found irrespective of the parameter settings of the module detection procedure''. Strong non-preservation is difficult to establish using cross-tabulation approaches that rely on module assignment in the test data set. A second disadvantage of a cross-tabulation based approach is that it requires that for each reference module one finds a matching test module. This may be difficult when a reference module overlaps with several test modules or when the overlaps are small. A third disadvantage is that cross-tabulating module membership between two networks may miss that the fact that the patterns of connectivity between module nodes are highly preserved between the two networks. The correlation network based statistics implemented in the modulePreservation function do not require the module assignment in the test network but require the user to input gene expression data underlying a reference data set and a test data set.

### Functional Enrichment Analysis

The Ingenuity Pathways Analysis (Ingenuity^®^Systems, http://www.ingenuity.com) software was used to determine whether sets of genes (e.g. preserved intramodular hub genes) were significantly enriched with known gene ontologies. This software ranks the pathways by their Fisher exact test p-value of functional enrichment. We chose the default background gene list (here all human genes) for the analysis. Ingenuity only reports uncorrected p-values. The gene lists published in our Additional files allow the reader to choose alternative backgrounds or software tools.

## Abbreviations

WGCNA: weighted gene co-expression network analysis; CD: cluster of differentiation; ME: module eigengene; MM: module membership; KME: eigengene-based connectivity.

## Competing interests

The authors declare that they have no competing interests.

## Authors' contributions

CC performed the research; CC, SH, TFF, PL, RL, and MCO helped analyze the data; CC and SH wrote the paper. RAO and LHB provided and guided the analysis of the Dutch blood data sets. SH designed the research. All authors read and approved the final manuscript.

## Supplementary Material

Additional file 1 **Description of pre-process of each data **Since data used in this manuscript are collected from different publications, each data used different platforms and different pre-process methods. This word document provides the detailed information of platform and pre-process methods which were provided by corresponding publications. Click here for file

Additional file 2 **Scatterplot of mean expression levels between brain and blood**. This pairwise scatterplots relates mean expression of 8799 expressed genes from the CTX, CN and CB brain data sets with corresponding genes from the Dutch and SAFHS blood data sets respectively. We report a robust estimate of the correlation coefficient (biweight midcorrelation, see method section). In each plot, uncorrected p-value (without brackets) is reported, as well as Bonferroni corrected p-value (with brackets). The extremely significant uncorrected correlation test p-values in scatterplots reflect the large sample size, i.e. numbers of genes. It may be more meaningful to focus on the correlation coefficient. Click here for file

Additional file 3 **Scatterplot of connectivity between brain and blood**. This pairwise scatterplots relates connectivity of 8799 expressed genes from the CTX, CN and CB brain data sets with corresponding genes from the Dutch and SAFHS blood data sets respectively. We report a robust estimate of the correlation coefficient (biweight midcorrelation, see the method section). We report both uncorrected and Bonferroni corrected p-values (inside the brackets). Click here for file

Additional file 4 **Evaluating the preservation of brain modules in blood**. This table reports the numeric preservation Z statistic for each brain module in both Dutch and SAFHS blood sets. Blue/CTX, green/CTX, yellow/CTX, yellow/CN and blue/CB modules consistently show significant preservation in both blood data sets. Click here for file

Additional file 5 **Cluster dendrogram of the blood data with different module annotations**. The cluster dendrogram show the module definition based on the blood data. The first color band underneath the dendrogram shows the module assignment in blood. The remaining color bands show module assignment (for the preserved modules) based on the different brain regions. Visual inspection of these dendrograms reveals that genes from the preserved brain modules tend to cluster together in the blood data, which confirms the results of the module preservation Z statistics. Click here for file

Additional file 6 **Table of pairwise correlations between preserved CTX modules**. Each preserved CTX module (yellow/CTX, blue/CTX and green/CTX) is represented by its eigengene. High positive or negative correlations between eigengenes suggest that the modules are indistinguishable. Correlations between the yellow/CTX, blue/CTX and green/CTX are reported. Click here for file

Additional file 7 **Module membership for preserved CTX modules**. This table reports the membership value for three preserved CTX modules (yellow/CTX, blue/CTX and green/CTX) in the CTX, Dutch and SAFHS datasets. The module membership (kME) reports the correlation between a gene expression profile and the module eigengene. Click here for file

Additional file 8 **Module membership for preserved CN module**. This table reports the membership value for the preserved CN module (yellow/CN) in the CN, Dutch and SAFHS datasets. The module membership (kME) reports the correlation between a gene expression profile and the module eigengene. Click here for file

Additional file 9 **Module membership for preserved CB module**. This table reports the membership value for the preserved CB module (blue/CB) in the CB, Dutch and SAFHS datasets. The module membership (kME) reports the correlation between a gene expression profile and the module eigengene. Click here for file

Additional file 10 **Pairwise correlations of combined blood module membership and two individual blood module memberships**. This table reports the pairwise correlations between the summary blood MM measure and the two individual blood MM measures for each preserved module (yellow/CTX, blue/CTX, green/CTX, yellow/CN and blue/CB). The table shows that the summary MM measure adequately represents the individual measures. Click here for file

Additional file 11 **Pairwise correlations of combined CTX module membership and three individual CTX module memberships**. This table reports the correlations between the combined CTX MM measure (summarized from three preserved CTX modules' module membership) and three individual CTX module memberships. The table shows that the combined CTX MM measure adequately represents the individual measures. Click here for file

Additional file 12 **Preserved hub genes list and their module membership values**. This table report three lists of preserved hub genes selected in Figure 5. Their corresponding module membership values are also provided. Click here for file

Additional file 13 **Results of an Ingenuity analysis for each preserved hub genes**. The table shows the Ingenuity functional enrichment for three preserved hub gene lists from Additional file 12 (only categories with p-value smaller than 1.0E-4 are shown). It also provides the detailed sub categories, as well as the corresponding genes. Click here for file

Additional file 14 **Relationship between modules and cluster of differentiation genes**. Table of correlation coefficients (and p-values) between cluster of differentiation genes and preserved module eigengene Click here for file

Additional file 15 **Annotation of cluster of differentiation genes that correlate with preserved modules**. Detailed functional annotation of significantly correlation cluster of differentiation genes selected from Additional file 14. Click here for file

Additional file 16 **Studying the brain modules preservation in other brain regions**. Here we report the results from applying the modulePreservation function to evaluate module preservation between brain regions. For example, we evaluate whether CTX brain modules are preserved in CN and CB regions. We also evaluate the preservation of CN modules (and CB) modules in the other regions. The bars in the barplots correspond to the preservation Z statistic of the modules. Each bar is colored by the original module color from Oldham et al. Click here for file

Additional file 17 **Scatterplot of mean expression levels and heritability**. This figure provides the scatterplot of mean expression level of top heritable genes with their heritability value. Uncorrected correlation test p-values and Bonferroni corrected p-values are reported (inside brackets). Click here for file
