# Supplementary material for: Is human blood a good surrogate for brain tissue in transcriptional studies?
Source: BMC Genomics. 2010 Oct 20;11:589. doi: 10.1186/1471-2164-11-589 (PMC3091510; doi:10.1186/1471-2164-11-589)
Supplement: Additional file 1 — Description of pre-process of each data Since data used in this manuscript are collected from different publications, each data used different platforms and different pre-process methods. This word document provides the detailed information of platform and pre-process methods which were provided by corresponding publications. [file 1471-2164-11-589-S1.DOC]

**Additional file 1**

**Description of microarray gene expression data and normalization methods.**

Here we provide more details regarding the gene expression and normalization methods. For the publicly available data downloaded from GEO, we adapt the description used in the original publications.

**SAFHS blood expression data**

The data and normalization steps are described in [1] but we provide the following terse description. Hybridization of RNA to Illumina Sentrix Human Whole Genome (WG-6) Series I BeadChips and subsequent washing, blocking and detection were carried out using Illumina’s BeadChip 6×2 protocol. Samples were scanned on the Illumina BeadArray 500GX Reader using Illumina BeadScan image data acquisition software (version 2.3.0.13). Illumina BeadStudio software (version 1.5.0.34) was used for preliminary data analysis, with a standard background normalization, to generate an output file for statistical analysis [1].

**Dutch blood expression data**

More details on the data can be found in [2]. Briefly, the Illumina Sentrix HumanRef-8 Expression BeadChip with >22,000 current RefSeq curated gene targets was used to obtain gene expression data. Cubic spline normalization was performed in Illumina's software package Beadstudio. After normalization data was imported into R http://www.r-project.org. About one third of the genes (8,000 genes) were found to be significantly expressed in peripheral blood at measurable levels (Bead studio mean detection level of p < 0.05). These genes were selected as starting point of the coexpression network analysis as described in [2].

**Cortex (CTX) brain expression data**

The CTX data combined publicly available data from three publications [3]. Details on the data can be found in [3]. Here we will briefly review the descriptions from the original publications.

The first publication used Affymetrix HU133A arrays. After hybridization, the signal on the chip was scanned using an HP GeneArray scanner and was processed by GeneSuite software (Affymetrix). Then the microarray raw data was processed by MAS5 (Affymetrix) and imported into GeneSpring 6.1 software (Silicon-Genetics, Redwood, CA, USA). Data normalization was then performed by dividing each microarray data set by its median value, using GeneSpring 6.1 software[4].

The second publication used both Affymetrix HU133A and HU133B arrays. Two procedures were used to assess array quality and remove outlier chips; arrays defined as outliers by either procedure were excluded from further analyses. PM/MM difference outlier algorithm implemented in the dChip software (<http://www.biostat.Harvard.edu/complab/dchip>) was applied. In addition, a quality assessment algorithm based on weights from robust regression models fits of gene expression was used to correct for chip and probe effects. For these robust regression models, outlier probes receive lower weight in the model fitting. Chips with aberrant patterns of low weights were excluded from further analyses [5].

The third publications also used Affymetrix U133A arrays. Isolated total RNAwas was carried through the Affymetrix preparation protocol19. Affymetrix Microarray Suite 5 (MAS5) was used for image processing and data acquisition and the raw data were then subjected to stringent quality control (QC) procedure. Expression measures were computed using the robust multi-chip average (RMA) method [6]

**CN and CB brain data**

Details are presented in [5]. Briefly, expression data was obtained by Affymetrix HU133A and HU133B. Two procedures were used to assess array quality and remove outlier chips; arrays defined as outliers by either procedure were excluded from further analyses. Again the PM/MM difference outlier algorithm of Li and Wong implemented in dChip software was used. In addition, quality assessment algorithm based on weights from robust regression models fits were used to correct for both chip and probe effects. For these robust regression models, outlier probes receive lower weight in the model fitting. Chips with aberrant patterns of low weights were excluded from further analyses [5].

**References**

1. Goring HH, Curran JE, Johnson MP, Dyer TD, Charlesworth J, Cole SA, Jowett JB, Abraham LJ, Rainwater DL, Comuzzie AG *et al*: Discovery of expression QTLs using large-scale transcriptional profiling in human lymphocytes. *Nat Genet* 2007, 39(10):1208-1216.
2. Saris CG, Horvath S, van Vught PW, van Es MA, Blauw HM, Fuller TF, Langfelder P, DeYoung J, Wokke JH, Veldink JH *et al*: Weighted gene co-expression network analysis of the peripheral blood from Amyotrophic Lateral Sclerosis patients. *BMC Genomics* 2009, 10:405.
3. Oldham MC, Konopka G, Iwamoto K, Langfelder P, Kato T, Horvath S, Geschwind DH: Functional organization of the transcriptome in human brain. *Nat Neurosci* 2008, 11(11):1271-1282.
4. Iwamoto, K., Bundo, M. & Kato, T. Altered expression of mitochondria-related genes in postmortem brains of patients with bipolar disorder or schizophrenia, as revealed by large-scale DNA microarray analysis. Hum. Mol. Genet 2005. 14, 241–253.
5. Hodges, A. et al. Regional and cellular gene expression changes in human Huntington’s disease brain. Hum. Mol. Genet 2006. 15, 965–977.
6. Ryan, M.M. et al. Gene expression analysis of bipolar disorder reveals downregulation of the ubiquitin cycle and alterations in synaptic genes. Mol. Psychiatry 2006. 11, 965–978.
