# Supplementary figures and images for: Is human blood a good surrogate for brain tissue in transcriptional studies?
Source: BMC Genomics. 2010 Oct 20;11:589. doi: 10.1186/1471-2164-11-589 (PMC3091510; doi:10.1186/1471-2164-11-589)

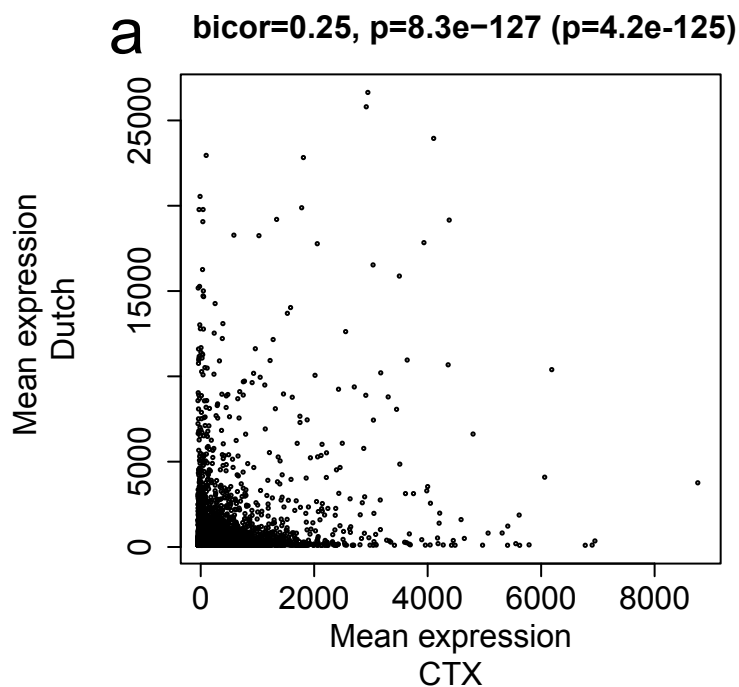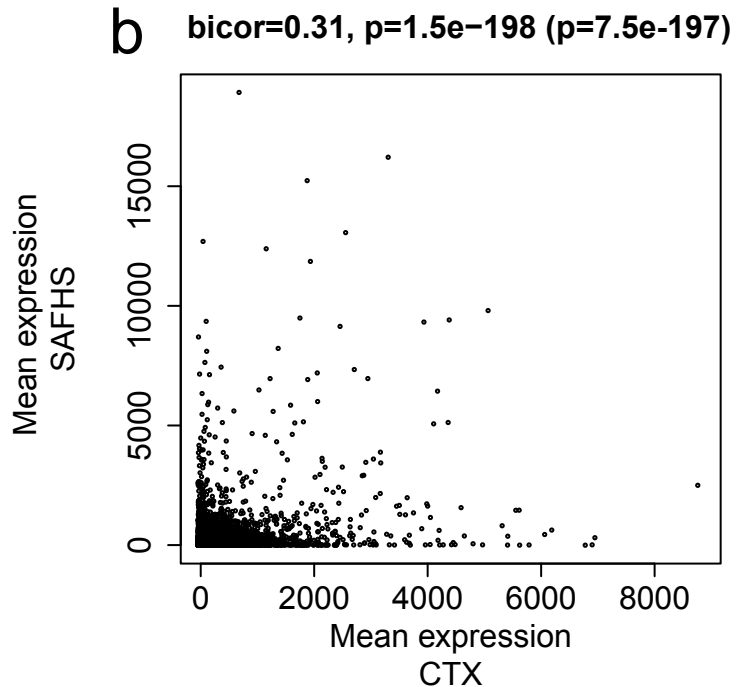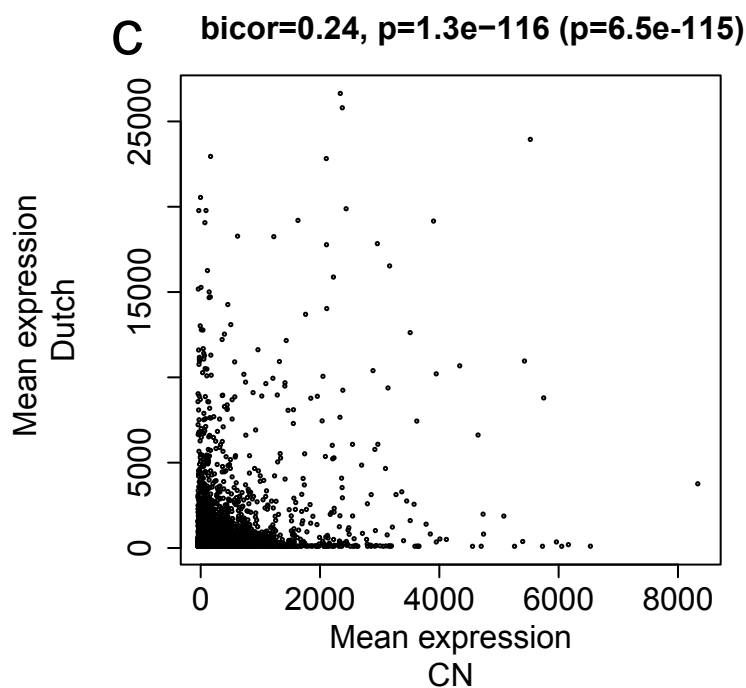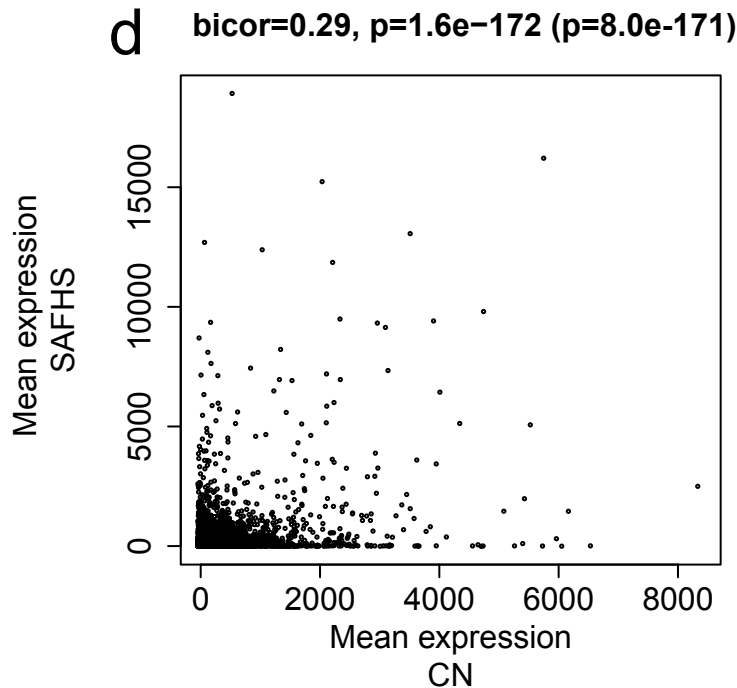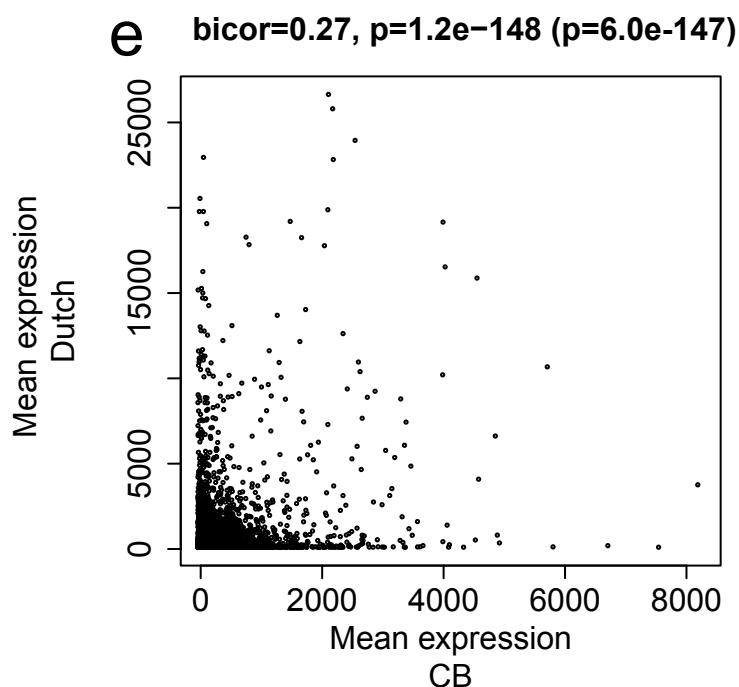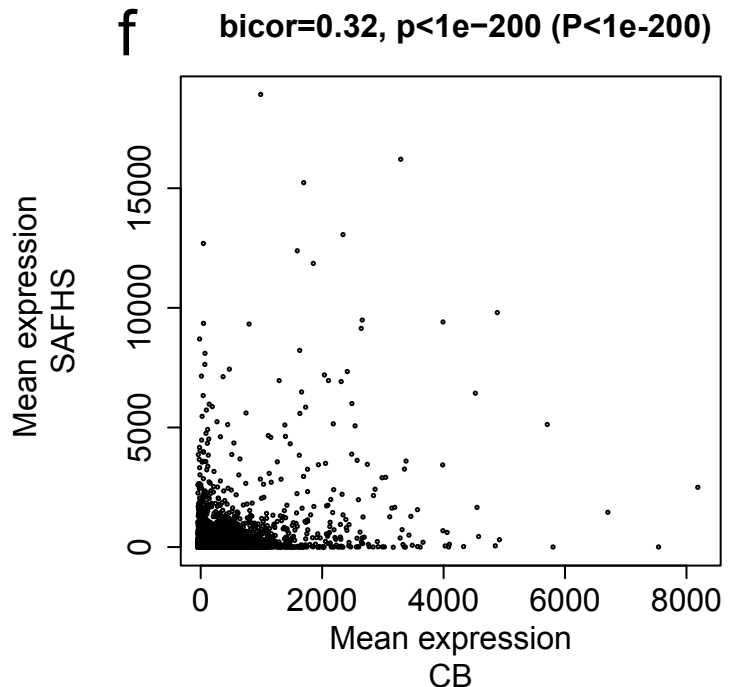

Supplement: Additional file 2 — Scatterplot of mean expression levels between brain and blood. This pairwise scatterplots relates mean expression of 8799 expressed genes from the CTX, CN and CB brain data sets with corresponding genes from the Dutch and SAFHS blood data sets respectively. We report a robust estimate of the correlation coefficient (biweight midcorrelation, see method section). In each plot, uncorrected p-value (without brackets) is reported, as well as Bonferroni corrected p-value (with brackets). The extremely significant uncorrected correlation test p-values in scatterplots reflect the large sample size, i.e. numbers of genes. It may be more meaningful to focus on the correlation coefficient. [file 1471-2164-11-589-S2.PDF]

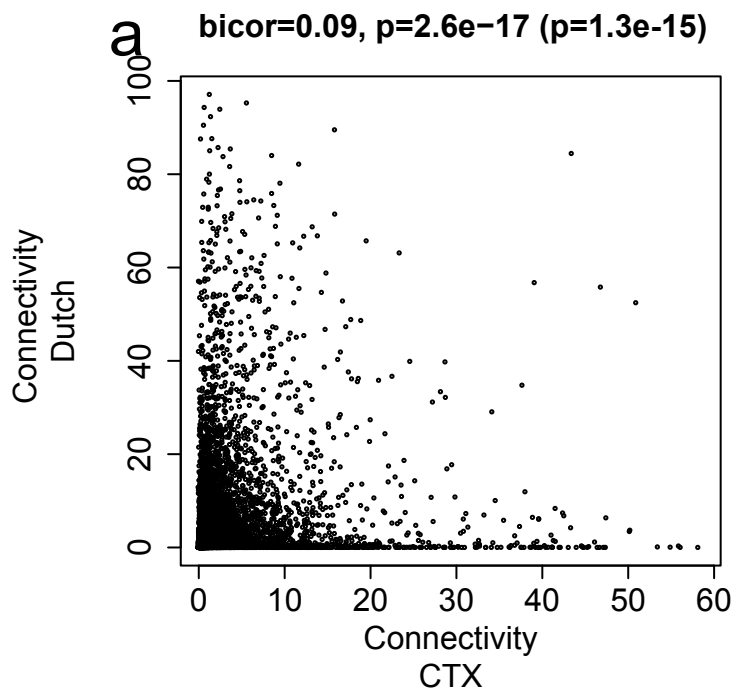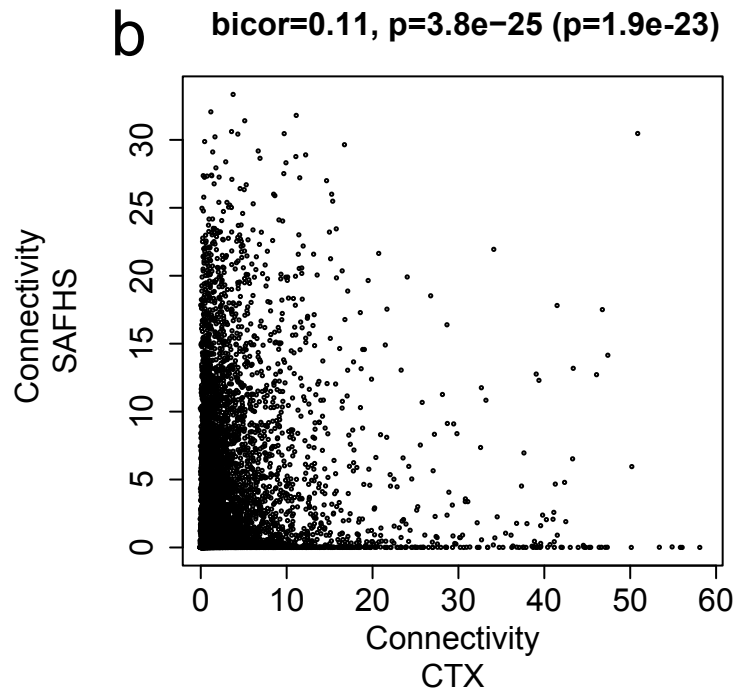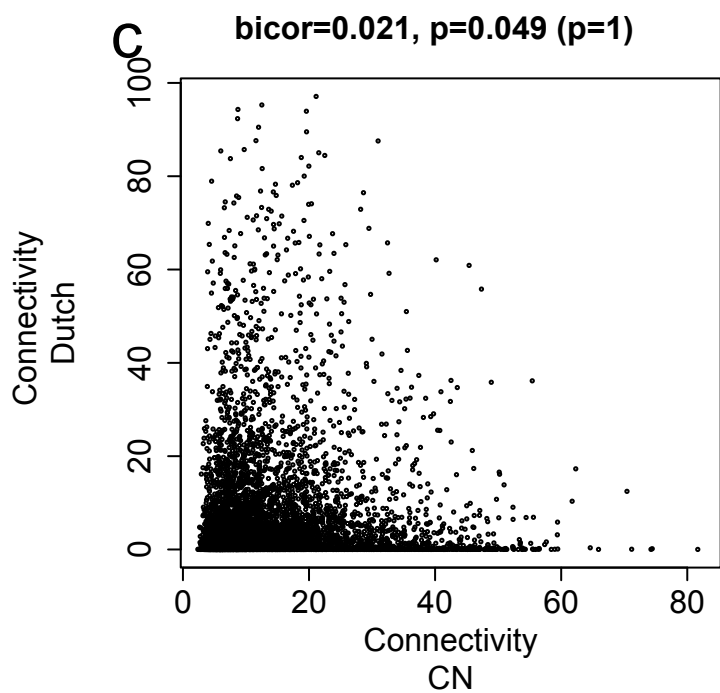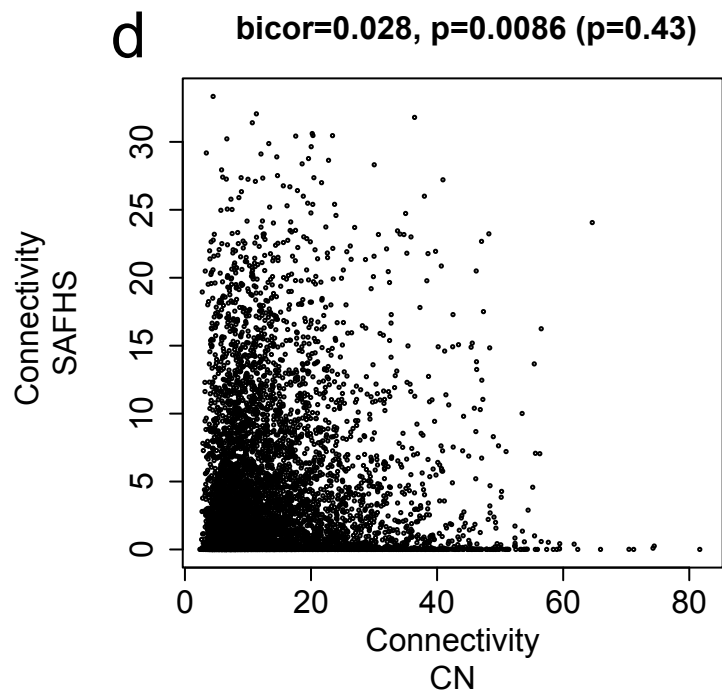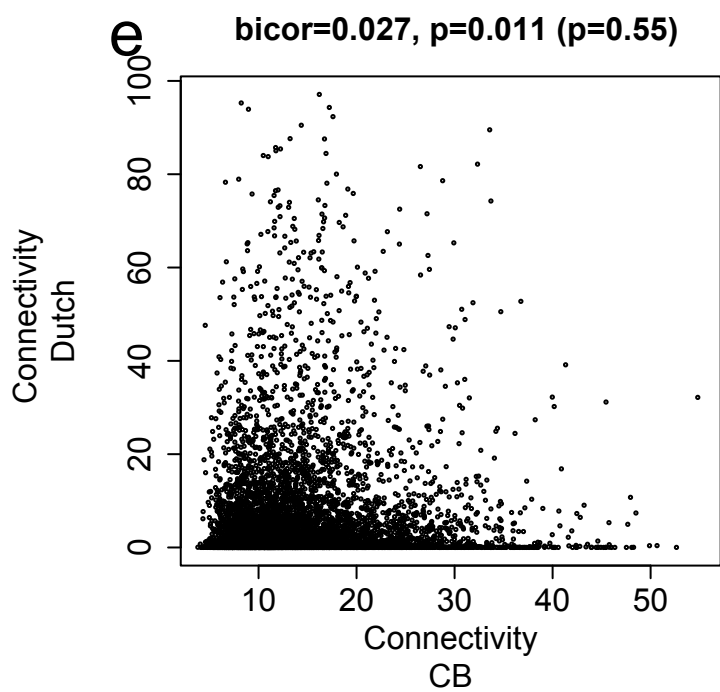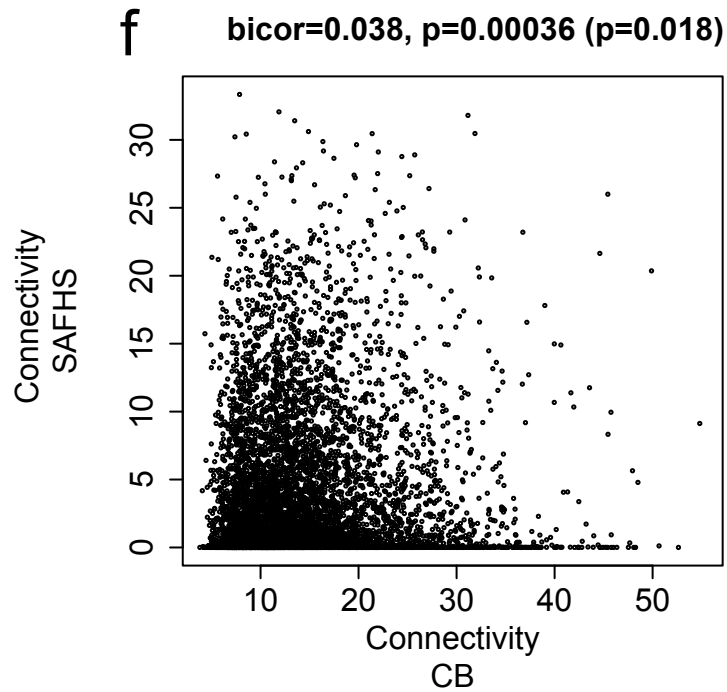

Supplement: Additional file 3 — Scatterplot of connectivity between brain and blood. This pairwise scatterplots relates connectivity of 8799 expressed genes from the CTX, CN and CB brain data sets with corresponding genes from the Dutch and SAFHS blood data sets respectively. We report a robust estimate of the correlation coefficient (biweight midcorrelation, see the method section). We report both uncorrected and Bonferroni corrected p-values (inside the brackets). [file 1471-2164-11-589-S3.PDF]

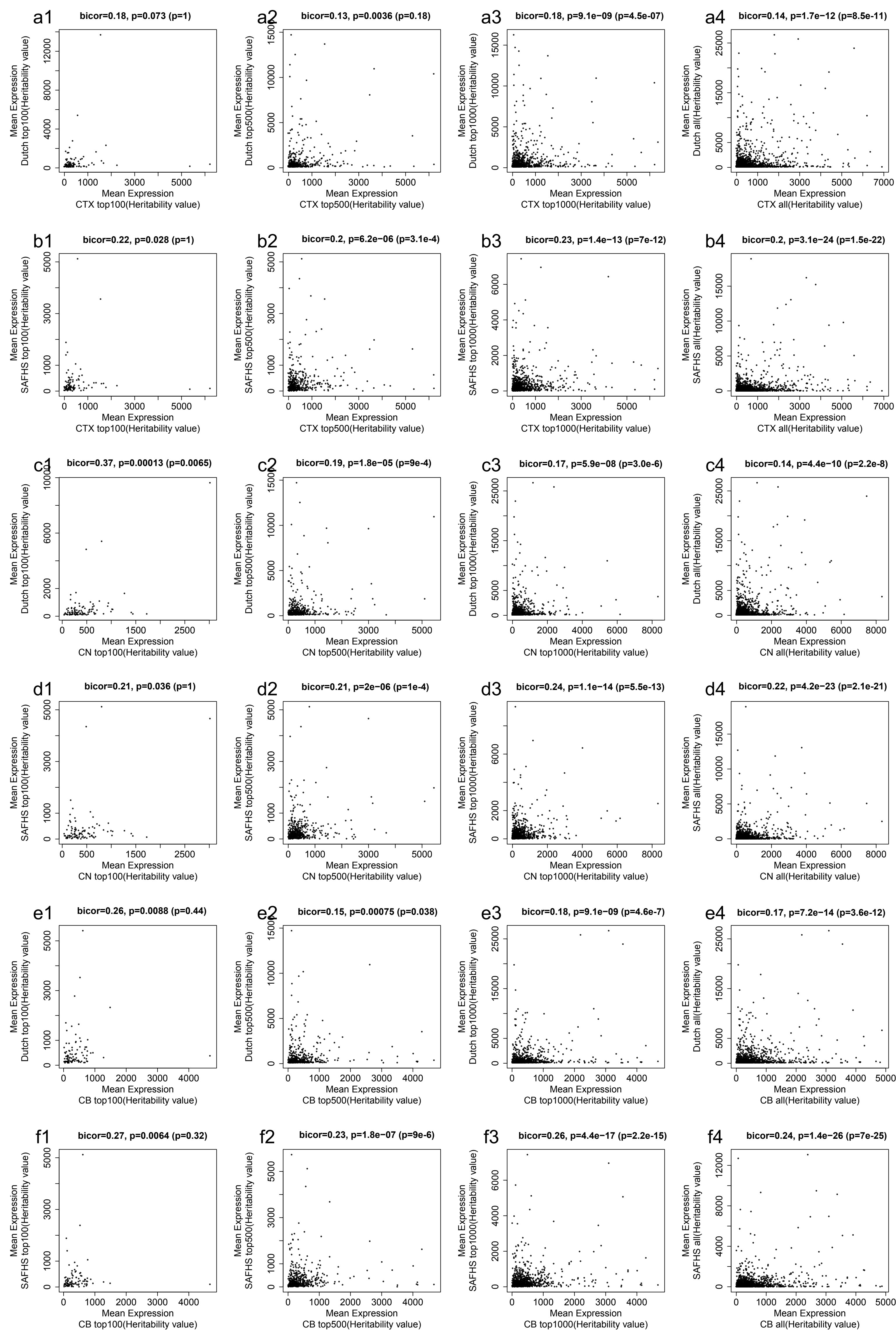

Supplement: Additional file 17 — Scatterplot of mean expression levels and heritability. This figure provides the scatterplot of mean expression level of top heritable genes with their heritability value. Uncorrected correlation test p-values and Bonferroni corrected p-values are reported (inside brackets). [file 1471-2164-11-589-S17.PDF]
