# Supplementary material for: Is human blood a good surrogate for brain tissue in transcriptional studies?
Source: BMC Genomics. 2010 Oct 20;11:589. doi: 10.1186/1471-2164-11-589 (PMC3091510; doi:10.1186/1471-2164-11-589)
Supplement: Additional file 5 — Cluster dendrogram of the blood data with different module annotations. The cluster dendrogram show the module definition based on the blood data. The first color band underneath the dendrogram shows the module assignment in blood. The remaining color bands show module assignment (for the preserved modules) based on the different brain regions. Visual inspection of these dendrograms reveals that genes from the preserved brain modules tend to cluster together in the blood data, which confirms the results of the module preservation Z statistics. [file 1471-2164-11-589-S5.PDF]

a

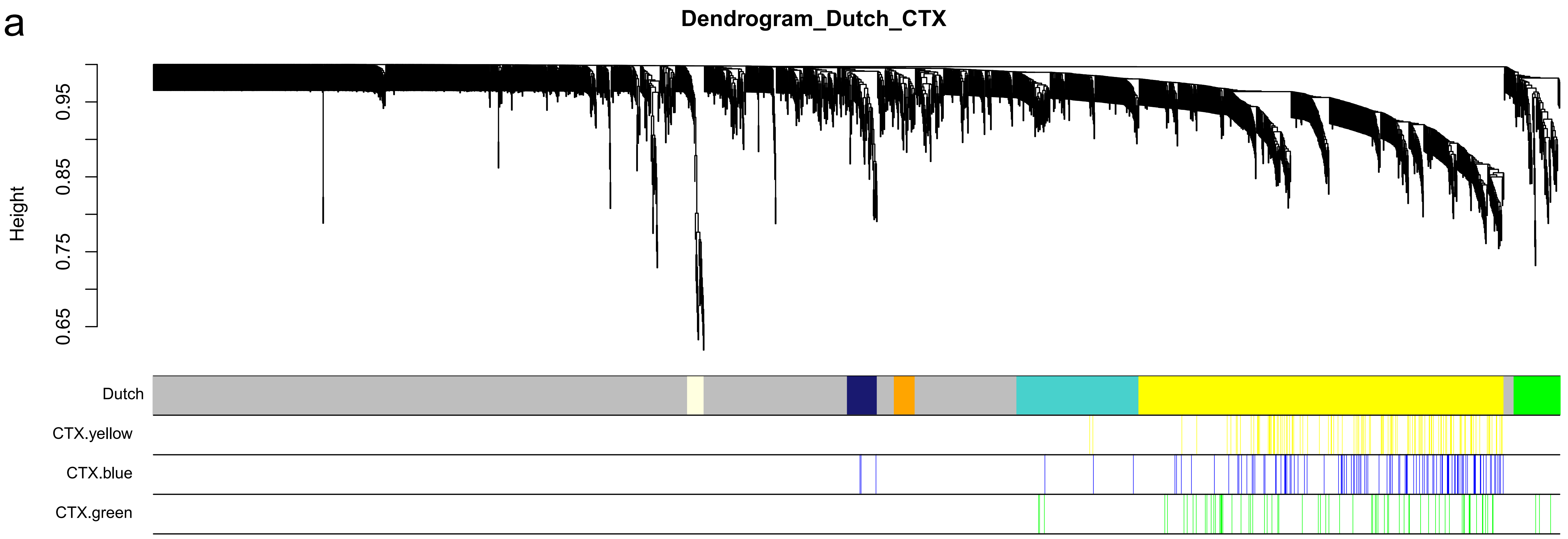

Dendrogram_Dutch_CTX
Height
0.95
0.85
0.75
0.65
Dutch
CTX.yellow
CTX.blue
CTX.green

b

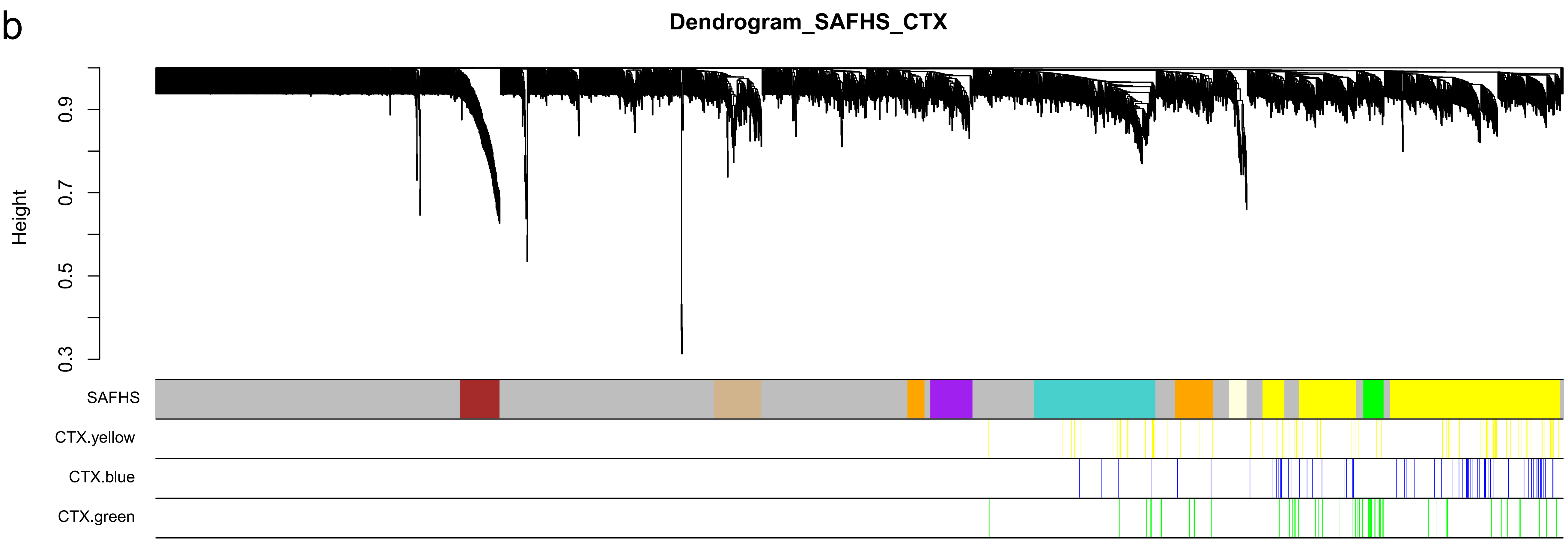
Dendrogram_SAFHS_CTX
Height
0.3
0.5
0.7
0.9
SAFHS
CTX.yellow
CTX.blue
CTX.green

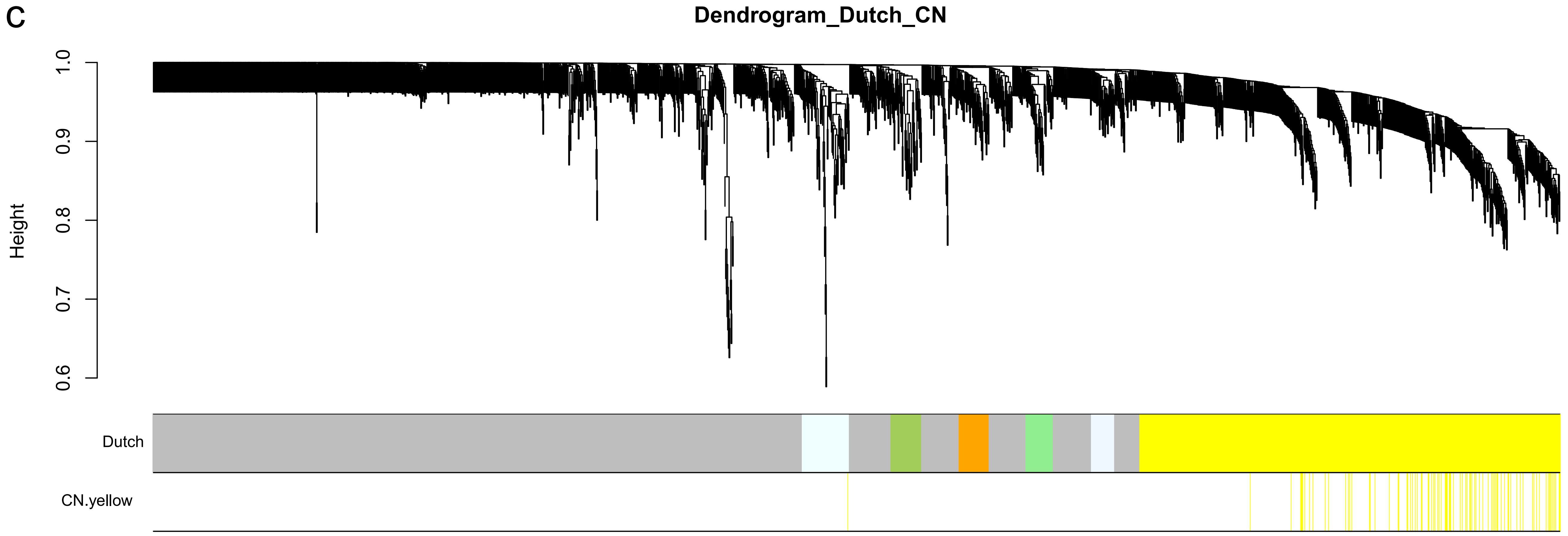
C
Dendrogram_Dutch_CN
Height
1.0
0.9
0.8
0.7
0.6
Dutch
CN.yellow

d

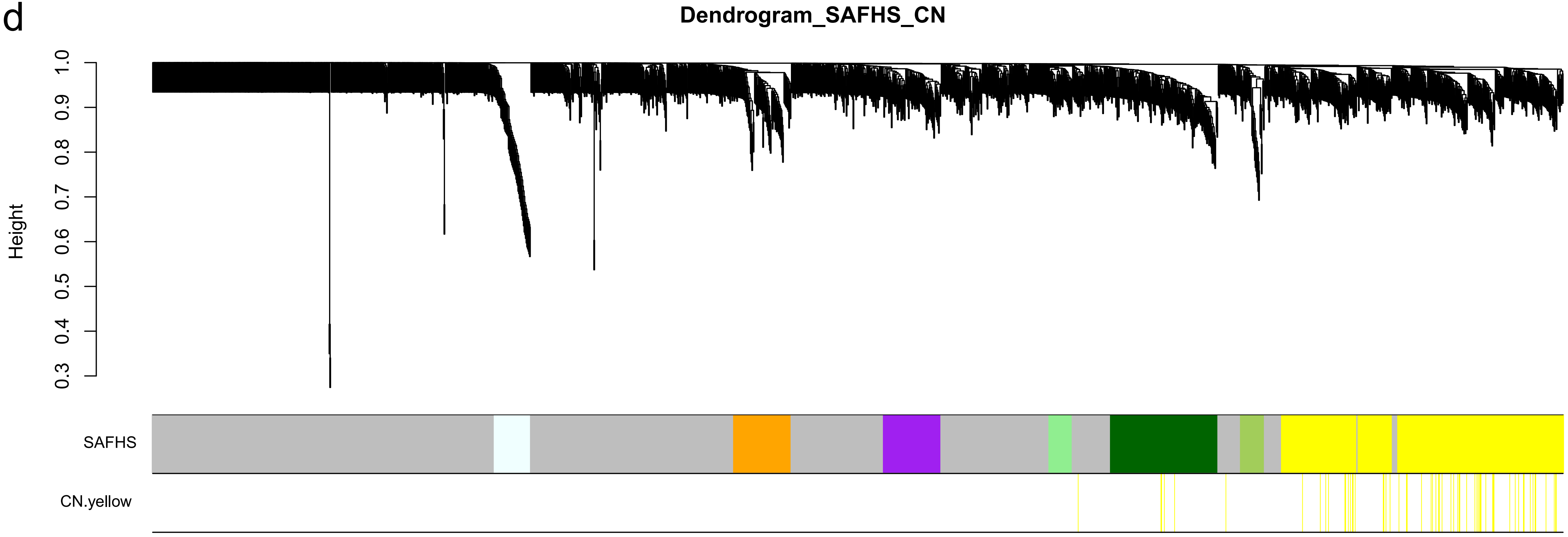
Dendrogram_SAFHS_CN
Height
1.0
0.9
0.8
0.7
0.6
0.5
0.4
0.3
SAFHS
CN.yellow

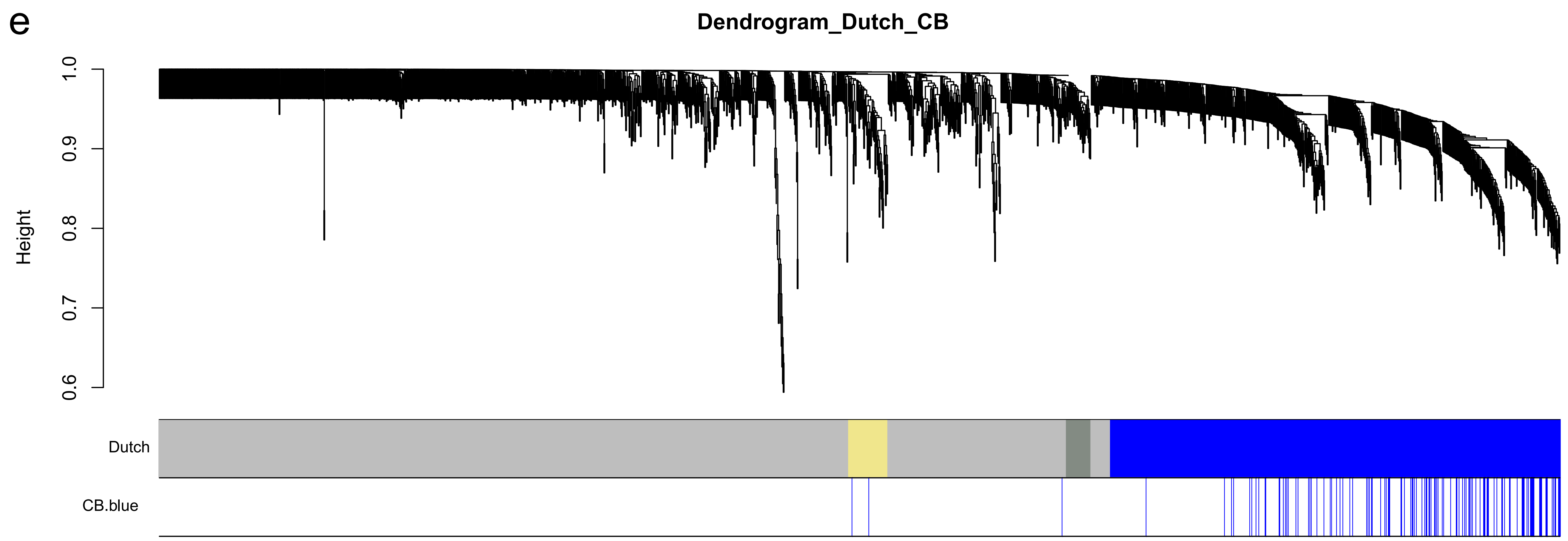
e
Dendrogram_Dutch_CB
Height
1.0
0.9
0.8
0.7
0.6
Dutch
CB.blue

f

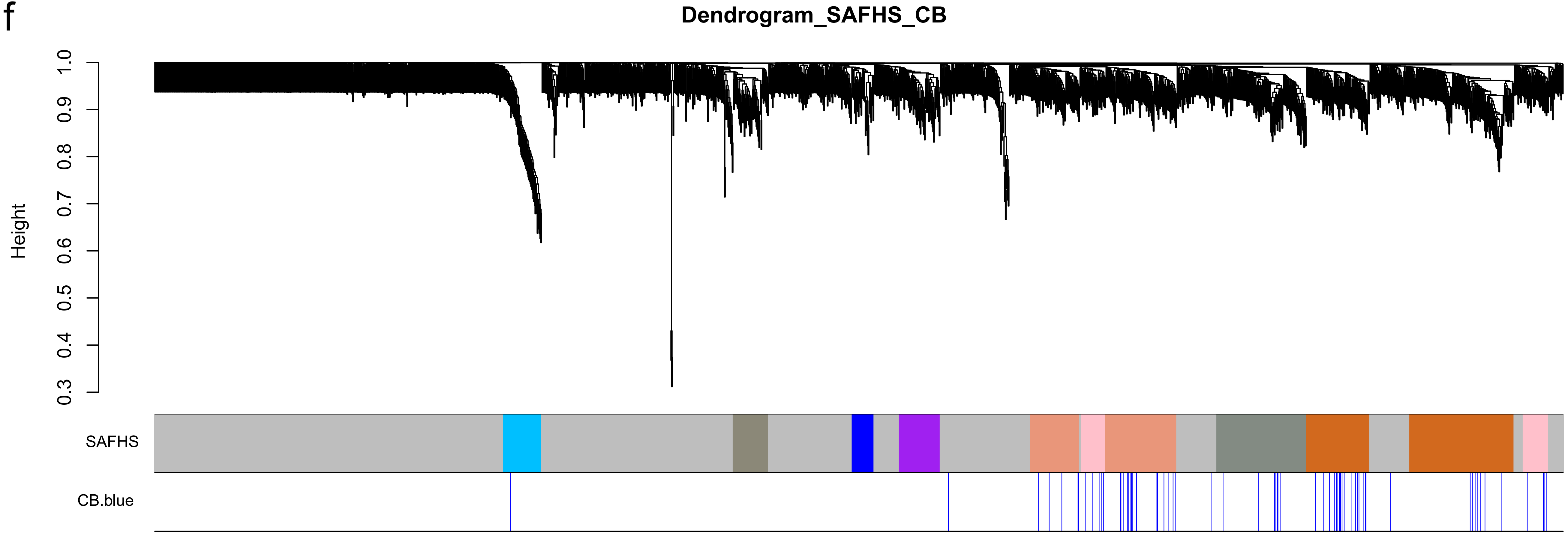
Dendrogram_SAFHS_CB
Height
1.0
0.9
0.8
0.7
0.6
0.5
0.4
0.3
SAFHS
CB.blue
