# Supplementary material for: Is human blood a good surrogate for brain tissue in transcriptional studies?
Source: BMC Genomics. 2010 Oct 20;11:589. doi: 10.1186/1471-2164-11-589 (PMC3091510; doi:10.1186/1471-2164-11-589)
Supplement: Additional file 16 — Studying the brain modules preservation in other brain regions. Here we report the results from applying the modulePreservation function to evaluate module preservation between brain regions. For example, we evaluate whether CTX brain modules are preserved in CN and CB regions. We also evaluate the preservation of CN modules (and CB) modules in the other regions. The bars in the barplots correspond to the preservation Z statistic of the modules. Each bar is colored by the original module color from Oldham et al. [file 1471-2164-11-589-S16.PDF]

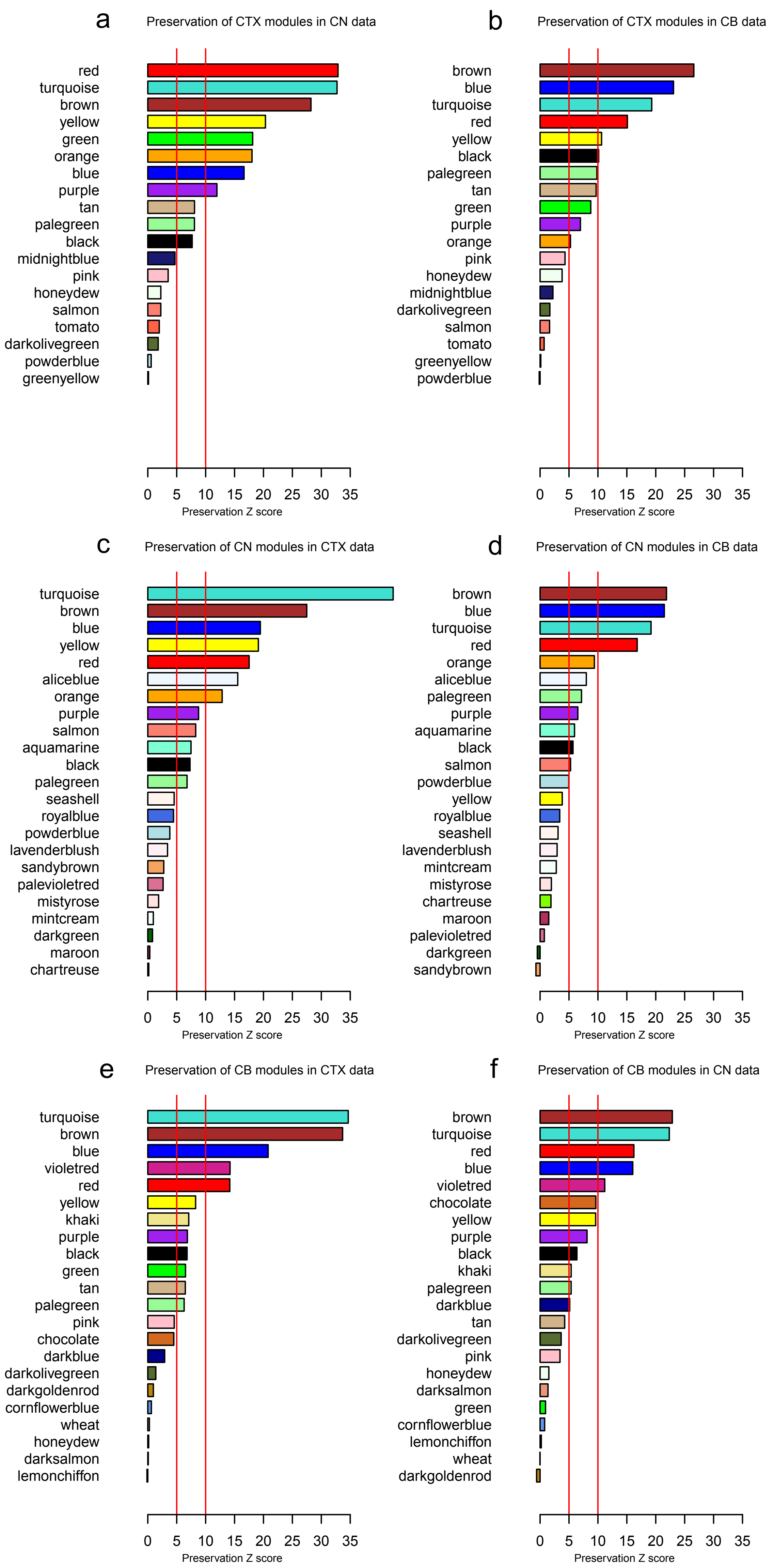
a
Preservation of CTX modules in CN data
red
turquoise
brown
yellow
green
orange
blue
purple
tan
palegreen
black
midnightblue
pink
honeydew
salmon
tomato
darkolivegreen
powderblue
greenyellow
0 5 10 15 20 25 30 35
Preservation Z score
b
Preservation of CTX modules in CB data
brown
blue
turquoise
red
yellow
black
palegreen
tan
green
purple
orange
pink
honeydew
midnightblue
darkolivegreen
salmon
tomato
greenyellow
powderblue
0 5 10 15 20 25 30 35
Preservation Z score
c
Preservation of CN modules in CTX data
turquoise
brown
blue
yellow
red
aliceblue
orange
purple
salmon
aquamarine
black
palegreen
seashell
royalblue
powderblue
lavenderblush
sandybrown
palevioletred
mistyrose
mintcream
darkgreen
maroon
chartreuse
0 5 10 15 20 25 30 35
Preservation Z score
d
Preservation of CN modules in CB data
brown
blue
turquoise
red
orange
aliceblue
palegreen
purple
aquamarine
black
salmon
powderblue
yellow
royalblue
seashell
lavenderblush
mintcream
mistyrose
chartreuse
maroon
palevioletred
darkgreen
sandybrown
0 5 10 15 20 25 30 35
Preservation Z score
e
Preservation of CB modules in CTX data
turquoise
brown
blue
violetred
red
yellow
khaki
purple
black
green
tan
palegreen
pink
chocolate
darkblue
darkolivegreen
darkgoldenrod
cornflowerblue
wheat
honeydew
darksalmon
lemonchiffon
0 5 10 15 20 25 30 35
Preservation Z score
f
Preservation of CB modules in CN data
brown
turquoise
red
blue
violetred
chocolate
yellow
purple
black
khaki
palegreen
darkblue
tan
darkolivegreen
pink
honeydew
darksalmon
green
cornflowerblue
lemonchiffon
wheat
darkgoldenrod
0 5 10 15 20 25 30 35
Preservation Z score
